# Sensory and Nutritional Profiling of Commercial Mussels (*Mytilus galloprovincialis* and *Mytilus edulis*) Across Different European Marine Origins

**DOI:** 10.3390/foods15173128

**Published:** 2026-09-03

**Authors:** Martina Magnani, Antonina De Marco, Matilde Cella, Asia Ferretti, Davide Nordio, Elisa Benini, Rais Urbini, Marina Silvi, Pier Paolo Gatta, Alessio Bonaldo

**Affiliations:** Department of Veterinary Medical Sciences, University of Bologna, Via Tolara di Sopra 50, Ozzano Emilia, 40064 Bologna, Italy; martina.magnani20@unibo.it (M.M.);

**Keywords:** Quantitative Descriptive Analysis, bivalve aquaculture, fatty acids, amino acid profile, umami taste, marine terroir

## Abstract

Mussels are a nutritionally valuable and environmentally sustainable seafood, yet how geographical origin and farming environment shape their sensory and nutritional quality remains insufficiently characterized across European production contexts. This study compared mussels from three contrasting European farming areas, Ireland (*Mytilus edulis*), Norway (*Mytilus edulis*) and Italy (*Mytilus galloprovincialis*), integrating Quantitative Descriptive Analysis (QDA^®^) with proximate composition, fatty acid, amino acid and mineral analyses. Italian mussels showed higher protein, lipid, carbohydrate and amino acid content together with the most intense and persistent umami taste, correlated with glutamic and aspartic acid content. Irish mussels were characterized by the highest moisture and iron content, associated with greater exudate release and salty/briny notes, while Norwegian mussels showed the lowest ash and amino acid content alongside greater bitterness, linked to omega-3 fatty acid content. These findings indicate that local environmental conditions shape origin-associated tendencies in the nutritional and sensory profile of farmed mussels, supporting the concept of a bivalve ‘marine terroir’ with potential applications for origin-based quality valorization.

## 1. Introduction

Among the major aquaculture species produced globally, sea mussels accounted for 1927 thousand tons (live weight equivalent) in 2022, ranking fourth in terms of total mollusk production [1]. Mussels are valued as a nutritious and sustainable seafood option [2,3]. From a nutritional standpoint, mussels are a remarkably complete food. They are an excellent source of high-quality protein, and compared to other animal protein sources, they are relatively low in calories and saturated fat, making them suited to balanced diets [4]. On the environmental sustainability front, mussels rank among the lowest impact animal foods: they require no feed as filter feeders, they actively improve water quality with their filtering action, and their carbon footprint is minimal [5].

The primary European mussel markets are concentrated in Southern EU countries, including Spain, France, Italy and Greece, where mussel consumption is high and exhibits a strong, localized demand, particularly in traditional coastal regions [6,7].

Italy, in particular, ranks as the second-largest mussel producer in Europe, yielding approximately 62,000 to 64,000 tons per year, primarily in the Northern Adriatic, and the predominant species farmed is *Mytilus galloprovincialis*, cultivated on rafts and longlines along most of Italy’s coastline [8]. Additionally, Italy is a major consumer of Mediterranean mussels, with significant seasonal variation in demand linked also to the nutritionally changing profile of the product [2]. Italian mussel consumption reached around 120,000 tons (live weight) in 2016, though this amount is supplied by both local aquaculture and imports, with Spain as the main supplier followed by Greece, Chile, and Tunisia filling the gap [9]. Despite this, Italian consumers tend to favor locally sourced mussels [10], widely consumed in coastal areas, where they remain an affordable seafood option [7,9].

In Ireland, mussel farming, primarily producing *Mytilus edulis* using rope or bottom culture methods, is a significant part of the shellfish sector [11,12]. In 2012, production totaled 15,000 tons [13]. Even if mussels are a key farmed shellfish in Ireland, the majority of production is exported, with relatively modest domestic consumption, which is reliant on efficient coastal logistics [2]. Irish consumers generally value the high quality and safety of locally farmed mussels [14].

While Ireland has an established mussel aquaculture industry with moderate production levels, Norway’s mussel farming sector is expanding, primarily focusing on blue mussels (*M. edulis*), with farms developing in fjordic environments, though it remains small compared to the more established finfish farming industry [11]. For Norwegian consumers, mussels are currently a minor food commodity, valued more for their environmental significance as indicators of contaminants and microplastics, rather than for high consumption volumes [15,16].

Across various countries, price and food safety remain major barriers to increased mussel consumption, while product quality is a key driver of demand [17,18,19], with appearance attributes such as meat color, along with nutritional quality, strongly influencing willingness to pay [20]. Nevertheless, given their sustainability and rich nutritional profile, mussels represent a low-cost and low-impact protein source, making them suited to meet the growing global demand for protein [21]. Mussels are rich in essential amino acids such as tryptophan and valine, with a favorable balance of saturated, monounsaturated, and polyunsaturated fatty acids, including significant levels of beneficial omega-3 polyunsaturated fatty acids (PUFAs) like EPA and DHA, which are advantageous for cardiovascular health [3,22]. They are also a good source of essential trace elements, including iron, zinc, selenium, and copper, which support metabolic functions and help address micronutrient deficiencies [23]. These nutritional characteristics significantly shape the sensory profile of mussels. Generally, sea mussels (*Mytilus* spp.) are characterized by a marine odor, umami and salty flavors, and a tender, juicy texture, with variations influenced by farming location and lipid/protein composition [24].

Environmental conditions at the farming site, such as temperature, salinity, and nutrient availability, along with farming practices, can strongly impact the nutrient composition, texture, aroma, visual appeal, and flavor of seafood [25,26]. For instance, variations in salinity can alter the texture (hardness, chewiness) and umami intensity in seafood species such as mud crabs (*Scylla paramamosain*) or largemouth bass (*Micropterus salmoides*) [27,28]. Similarly, elevated temperatures can reduce the concentration of amino acids and amines associated with sweet or bitter notes, thereby altering the aroma profile in species like blue swimmer crab (*Portunus armatus*) [29], and can affect the energy reserves in *M. galloprovincialis*, as well as protein, lipid, carbohydrate, caloric, and mineral content in oysters *Magallana gigas* and *Ostrea edulis*, and other bivalves [30,31,32,33].

Taken together, these findings suggest that the environmental conditions experienced during farming can contribute to shaping the nutritional, biochemical, and sensory characteristics of mussels, potentially generating site-specific signatures. This perspective provides a natural link to the concept of terroir, traditionally used to describe the influence of place and environment on the characteristics of agricultural products. Beyond its well-established application to viticulture [34], the terroir framework has begun to find precedent in bivalve aquaculture research: geographical origin has been shown to drive the biochemical composition and volatile/flavor profile of farmed mussels, as seen for *M. galloprovincialis* from three different Spanish origins [35]. Moreover, sensory panels have been able to discriminate mussels according to production site, as in the study of Oliveira et al. (2015) on mussels from Spanish and Portuguese production areas [36], and origin-related compositional signatures, investigated by near-infrared spectroscopy (NIRS), for example, have supported traceability and authentication approaches [10]. While the term ‘marine terroir’ itself has not yet been formally established within the peer-reviewed scientific literature, and remains largely confined to popular and trade publications, it offers a compelling conceptual lens that merits deeper scientific investigation, particularly for comparative studies across contrasting farming environments. In this context, the present study explores whether the ’marine terroir‘ concept applies to mussels reared under different European environmental conditions.

The present study aims to evaluate and compare the organoleptic and sensory characteristics of mussels (*M. galloprovincialis* and *M. edulis*) from three European farming regions, Italy, Ireland, and Norway. These countries were selected as case studies because they represent three contrasting yet complementary profiles in terms of mussel production and consumption. Italy embodies a long-standing Mediterranean maritime tradition in which mussels are deeply embedded in coastal gastronomy. In Ireland, despite its geographical relationship with the Atlantic Ocean, domestic mussel consumption plays a secondary role to export markets. Norway is a nation globally renowned for its seafood industry, but mussel farming remains marginal. Together, these three countries capture a broad spectrum of environmental conditions and production traditions, making them particularly suited to explore the diversity of mussel characteristics across Europe. This research seeks to understand how environmental and farming conditions influence the nutritional properties and, consequently, the sensory and commercial quality of the final product, examining the relationship between geographical origin and sensory profile.

## 2. Materials and Methods

### 2.1. Products and Experimental Design

Diploid farmed mussels from three European countries of origin, Ireland, Norway and Italy, were tested and compared in the study. Specifically, a batch of 8 kg of commercial blue mussels (*M. edulis*) was collected by the Irish Roaring Water Bay company (Skibbereen, West Cork, Ireland) (51°31′30″ N, 9°26′31″ W) and the Norwegian Norgeskjell AS company (Åfjord, Trøndelag, Norway) (63°56′33″ N, 10°11′04″ E), while 8 kg of Mediterranean mussels, *M. galloprovincialis* were collected by the Italian Emilia-Romagna Mussel Farmers’ Consortium in the North Adriatic sea (Cesenatico, Forlì-Cesena, Italy) (44°19′33″ N, 12°55′40″ E), where the product has been awarded the local “Romagnola Mussel” certification. The batches from the three locations were harvested during the same second week of July 2025, corresponding to the summer season. Then, the samples were shipped under refrigerated conditions to the Sensory Laboratory of the Department of Veterinary Medical Sciences, Alma Mater Studiorum, University of Bologna (UNIBO). The cold chain was maintained throughout transport and handling, with the samples kept in polystyrene boxes containing ice packs. The samples were analyzed immediately upon arrival at the laboratory. A sample of fifteen specimens from each site of origin was measured for the biometric parameters of whole specimen weight, soft tissue weight using a Radwag WLC balance (±0.1 g) (Radwag, Radom, Poland), and length using a digital caliper (±0.01 mm precision) (Metrica, Milan, Italy), and the condition index was calculated (CI = wet soft tissue weight (g)/whole specimen weight (g) × 100) [37].

Environmental conditions of the rearing areas were considered in order to contextualize and support the comparison of sensory and nutritional profiles of mussels from the three different origins. Specifically, environmental data were acquired for the twelve-month period immediately preceding mussel harvesting (July 2024–July 2025), corresponding to the full farming cycle leading up to the collection of the specimens subsequently used for sensory and biochemical analyses. Key oceanographic variables, including seawater temperature, salinity, chlorophyll concentration (used as a proxy for phytoplankton abundance and thus food availability for mussels), and current velocity, were retrieved from the Copernicus Marine Service dataset [38,39]. These parameters were used to characterize the environmental conditions potentially influencing mussel quality during the farming cycle.

### 2.2. Sensory Evaluation

Sensory evaluation was performed by a trained panel of six assessors (mixed gender, aged 25–45 years) recruited internally from the University Department of Veterinary Medical Sciences (PhD students, technicians and researchers), with an academic/research background in aquaculture and food science and at least two years of continuous experience in sensory panel work. Panel selection and training were carried out according to ISO 8586:2023 [40] and included screening for olfactory and gustatory acuity, recognition of basic odors and tastes, and familiarization with the sensory properties of the product category. Training comprised weekly sessions over a three-month period and included calibration against reference standards (e.g., aqueous solutions for the basic tastes: acid, sweet, salty and bitter) and against mussel samples for product-specific descriptors such as briny and umami.

Descriptor generation, selection and standardization were established according to the principles of sensory profiling described in ISO 11035:1994 [41] and in descriptive sensory methodology references [42,43,44]. An initial pool of candidate descriptors was compiled from established vocabularies for the sensory evaluation of seafood and bivalves, including the Codex Alimentarius guidelines for the sensory evaluation of fish and shellfish [45], standardized freshness/quality score sheets for fish and shellfish, used as a source vocabulary [46], and descriptive sensory studies on mussels [47,48]. This pool was subsequently refined through panel discussion and consensus into the final list of 21 descriptors, reported in Table 1 with their definitions.

Before assessment, mussels were shelled and placed in transparent glass jars to allow visual evaluation. The jars containing the samples were then cooked in a conventional oven at 115 °C for 10 min. Immediately after cooking, samples were kept warm until serving to the assessors in order to preserve serving temperature and aroma release. Sensory sessions were carried out in individual booths in a tasting room designed according to ISO 8589:2007/Amd 1:2024 [49]. Within each session, samples were presented in a balanced order to minimize order and first-order carry-over effects, following the principles described by Macfie et al. in 1989 [50]. Water was provided between samples as a palate cleanser.

Sensory evaluations were conducted over three sessions. In each session, all three origins were evaluated by the trained assessors. Quantitative Descriptive Analysis (QDA^®^) was conducted following the general principles for establishing a sensory profile described in ISO 13299:2016 [51]. The method was used to characterize the samples across the domains of odor, appearance, flavor, basic tastes, texture, aftertaste and mouthfeel. Each descriptor was rated on an unstructured 10 cm linear scale anchored at the extremes, corresponding to absence of the attribute (0) and maximum perceived intensity (10).

### 2.3. Nutritional Analysis

The soft body of mussels was separated from the shell to obtain about 500 g of pulp per country. Then the pulp was homogenized to get a homogeneous sample mass.

The analyses were performed in duplicate.

The moisture was determined by drying a portion of the sample at 105 °C in a Binder FD 115 oven (Binder GmbH, Tuttlingen, Germany) until a constant weight was reached.

For the determination of proteins, the sample was weighed in a crucible and placed in an Elementar Rapidmax N (Elementar Italia, Como, Italy) nitrogen analyzer equipped with a combustion tube at a temperature of 920 °C, where the nitrogen compounds were converted to molecular nitrogen and determined quantitatively by a thermal conductivity detector. Then, nitrogen was transformed into proteins by a conventional multiplication factor of 6.25.

Fat content was quantified by petroleum ether extraction using a Soxhlet system for 4 h, followed by drying the residue at 100 °C in a Binder FD 260 oven (Binder GmbH, Tuttlingen, Germany).

To determine the ash, a known quantity of the sample was weighed in a capsule (5–10 g), calcined and incinerated in a Falc FM 22 muffle (Falc Instruments, Bergamo, Italy) at 550 °C. The temperature was maintained until the organic substance was completely burnt and the ash appeared light gray/white. The capsule was placed in a Binder FD 240 oven (Binder GmbH, Tuttlingen, Germany) at 103 °C and weighed.

The carbohydrate concentration was calculated by deducing from 100 the values of proteins, fat, ash and moisture, a standard approach in bivalve proximate composition analysis [52,53]. As this indirect approach cumulates the analytical errors associated with the four preceding proximate determinations, the resulting values are reported as calculated total carbohydrate equivalents rather than as a direct, validated quantification of carbohydrate or glycogen content. This limitation should be considered when interpreting the absolute carbohydrate values reported in this study, particularly for the Italian samples, in which higher protein and lower moisture content may proportionally amplify the cumulative error of the by-difference calculation.

For the calculation of energy value, expressed in both Kcal and KJ, the energy components of the food were multiplied by specific factors (4 kcal/g and 17 kJ/g for carbohydrates and protein respectively; 9 kcal/g and 37 KJ/g for fat) and then added together [54].

Selenium, iron, zinc and copper contents were determined by inductively coupled plasma (ICP-MS) Thermo Scientific iCAP TQe (Thermo Fisher Scientific, Bremen, Germany); phosphorus, sodium, potassium and magnesium were determined by inductively coupled plasma optical emission spectroscopy (ICP-OES) Thermo Scientific iCAP-PRO (Thermo Fisher Scientific, Bremen, Germany) after acidic digestion of the sample (Microwave MARS6 CEM or Ultrawave Milestone, Milestone, Bergamo, Italy) and expressed in mg/kg.

The composition of the fatty acids was analyzed by an Agilent Technologies 7890A gas chromatograph equipped with a 7693 autosampler and a flame ionization detector (GC/FID) (Agilent Technologies, Milan, Italy).

Amino acid composition was determined using a Biochrom 30+ Amino Acid Analyzer instrument equipped with an ion exchange column, post-column ninhydrin derivatization and a UV–visible detector (Biochrom Ltd., Cambridge, United Kingdom).

For moisture, protein, fat, ash, carbohydrate, fatty acid and amino acids, the quantities were reported in percentage, i.e., g/100 g.

### 2.4. Statistical Analysis

Data related to biometric parameters (whole specimen weight, soft tissue weight, length and condition index), each sensory descriptor attribute and each nutritional parameter were analyzed using one-way analysis of variance (ANOVA), followed by Tukey’s post hoc test when significant differences among geographical origins were detected. Statistical significance was set at *p* < 0.05.

To obtain an overall multivariate representation of the sensory dataset, Principal component analysis (PCA) was performed on the sensory dataset, assessing the differences between mussel products. Euclidean distances were calculated to determine the differences between the centroids representing the average positions of each treatment group in the multidimensional space. In the resulting PCA plot, descriptor attributes with a Pearson correlation of ≥75% were represented as overlapping vectors.

To further explore potential relationships between sensory and nutritional traits, Pearson’s correlation coefficients were calculated between the sensory descriptor attributes and the nutritional parameters, using the geographical origin mean values (Italy, Ireland, Norway; *n* = 3) as observations. Results were visualized as a correlation heatmap, with color intensity representing the magnitude and sign of the Pearson correlation coefficient (r). Given the limited number of geographical origins, these correlations should be regarded as descriptive and exploratory tools to highlight potential macro-trends rather than statistically inferential. Moreover, due to the large number of nutritional variables measured, a subset of nutritional parameters was selected for the correlation analysis, comprising proximate composition, the main fatty acid fractions (SFA, MUFA, PUFA, omega-3, omega-6, omega-9) together with fatty acids of specific nutritional or metabolic relevance (e.g., EPA, DHA, 18:1 n-7, 18:4 n-3), selected amino acids associated with taste-active compounds (aspartic acid, glutamic acid, alanine, glycine, histidine, threonine), and minerals showing significant differences among origins or biological relevance to the sensory traits discussed (iron, phosphorus and zinc).

All the statistical analyses were carried out using GraphPad Prism (version 10; GraphPad Software, San Diego, CA, USA).

## 3. Results

The mussels sampled at the three origin sites showed essentially comparable biometric values (Table 2). The weight of the whole specimen ranged from 16.05 ± 1.01 g (Ireland) to 17.97 ± 1.23 g (Italy), with Norway falling in between (16.95 ± 2.94 g). A similar trend was observed for the weight of the edible portion or soft tissue (6.32–7.16 g) and for shell length (5.95–6.39 cm), while the condition index ranged from 39.30% (Ireland) to 40.59% (Norway). Despite the slight numerical differences observed among the sites, statistical analysis did not reveal any significant differences among the origins for any of the biometric parameters considered (*p* > 0.05).

The analysis of the Copernicus Marine dataset indicated that the farming areas of the three mussel origins were characterized by distinct environmental conditions over the considered period (July 2024–July 2025) (Table 2). Water temperature showed the widest range at the Italian site (7.98–30.2 °C), whereas the Irish and Norwegian sites exhibited more moderate thermal fluctuations. Salinity ranged between 29.5 and 36.5 across all three sites, with slightly higher values recorded in the Adriatic. Maximum current velocity was highest at the Ireland site (0.444 m/s), followed by Norway and Italy. Finally, chlorophyll-α concentration showed slightly higher peaks at the Italian site (0.235–3.37 mg/m^3^) relative to the Irish and Norwegian sites.

**Figure 1:**
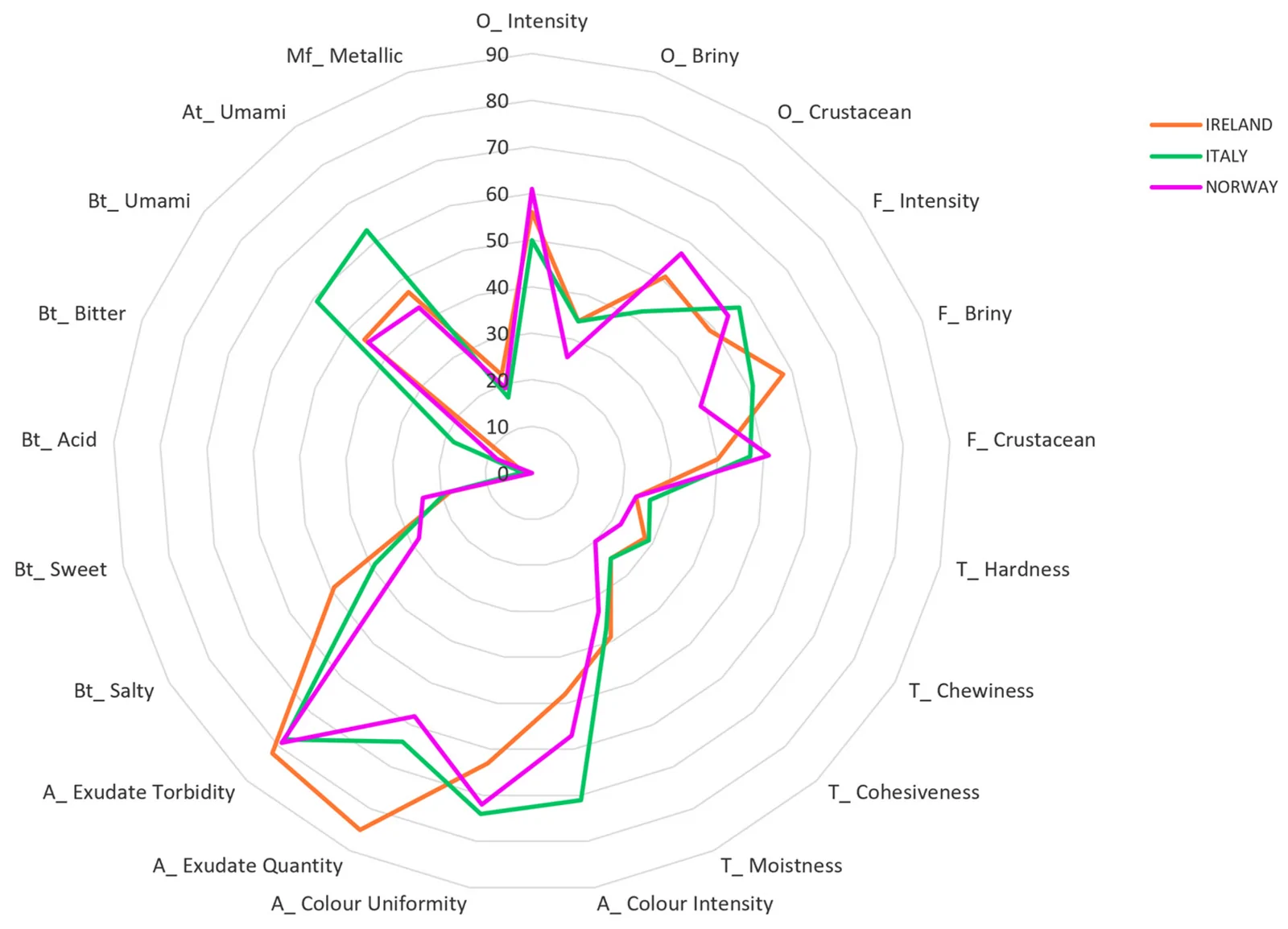
Complete sensory profile of mussels from Ireland, Norway and Italy. From right to left: O = odor, F = flavor, T = texture, A = appearance, Bt = basic taste, At = aftertaste, Mf = mouthfeel.

The sensory analysis conducted on mussels originating from Ireland, Norway, and Italy revealed marked statistically significant differences among the three groups (Figure 1).

Norwegian mussels were characterized by a higher odor intensity than Italian mussels (*p* = 0.030), whereas Irish mussels showed an intermediate value and did not differ significantly from either origin (*p* > 0.05).

Briny flavor was significantly higher in Irish than in Norwegian mussels (*p* = 0.014), while Italian samples showed an intermediate value.

Regarding appearance, color intensity was significantly higher in Italian than in Irish mussels (*p* = 0.021), with Norwegian mussels showing an intermediate value. Exudate quantity was significantly higher in Irish than in Norwegian mussels (*p* = 0.020), whereas Italian mussels were intermediate.

Among basic tastes, salty taste was significantly higher in Irish and Italian mussels than in Norwegian mussels (*p* < 0.001 and *p* = 0.002, respectively). Norwegian mussels were perceived as significantly more bitter than Irish mussels (*p* = 0.040), while Italian samples showed an intermediate value. Italian mussels had significantly higher umami intensity than Norwegian mussels (*p* = 0.045), with Irish samples showing an intermediate value. A similar pattern was observed for umami aftertaste, which was significantly higher in Italian than in Norwegian mussels (*p* = 0.014), while Irish mussels were intermediate.

No significant differences among geographical origins were observed for briny odor, crustacean odor, flavor intensity, crustacean flavor, color uniformity, exudate turbidity, hardness, chewiness, cohesiveness, moisture, sweetness, acidity, or metallic mouthfeel (*p* > 0.05). The complete set of mean values, standard deviations and post hoc *p*-values for all 21 sensory descriptors is provided in Supplementary Table S1.

Principal component analysis performed on the complete raw dataset (54 observations, 21 sensory variables) identified PC1 and PC2 as the first two components, explaining 19.01% and 12.93% of the total variance, respectively (31.94% cumulative). Eight principal components were required to reach 75% of the cumulative variance, indicating that the sensory variability pattern of the dataset is distributed across a relatively large number of independent dimensions, highlighting a substantial intra-group variability, as expected for complex sensory data matrices containing numerous variables that are correlated with one another in a non-linear manner.

**Figure 2:**
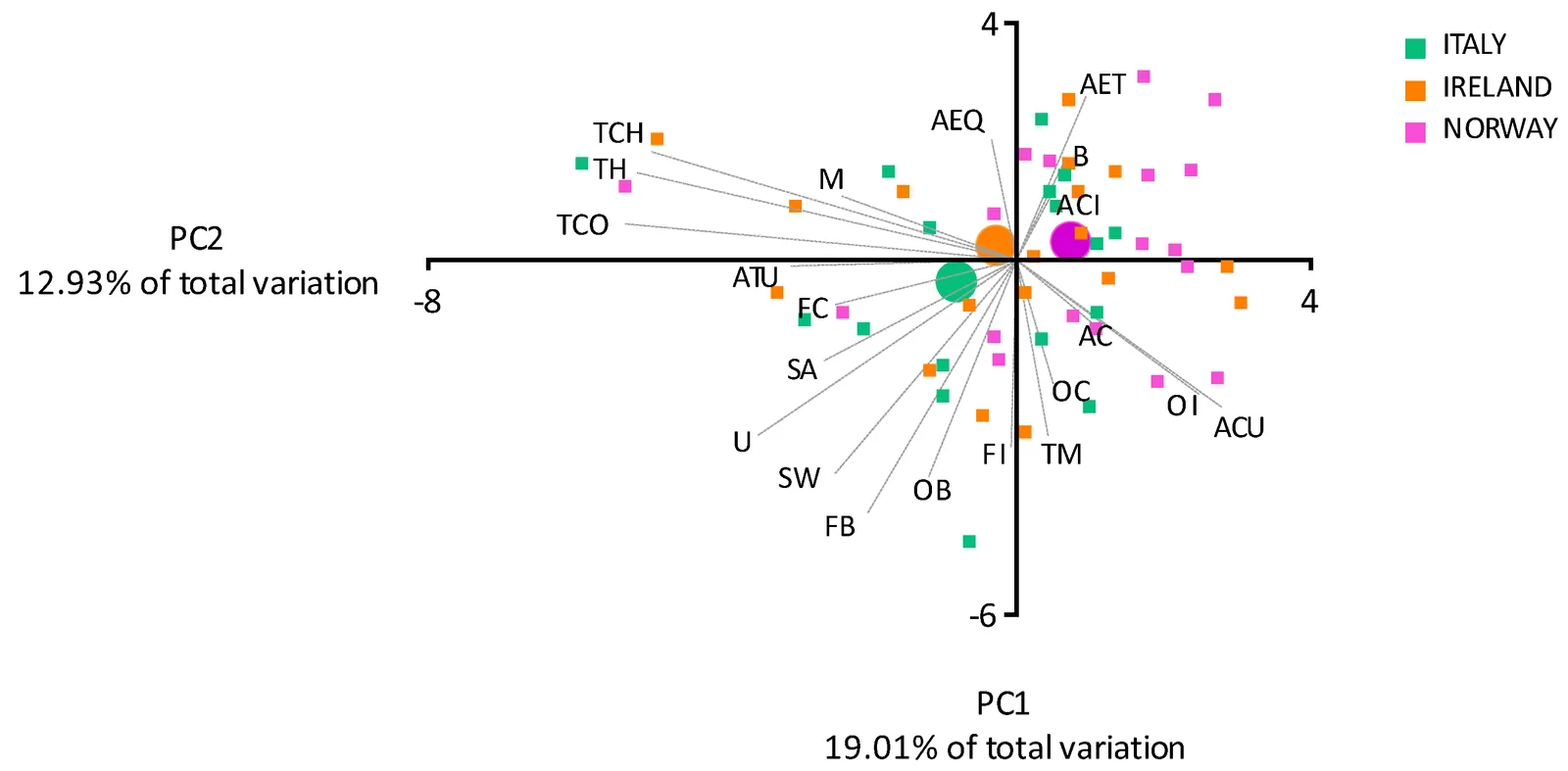
Principal component analysis (PCA) biplot of the complete sensory dataset of mussels from Ireland, Norway and Italy. Individual points represent sensory evaluations, whereas vectors indicate the contribution of each sensory descriptor attribute to the first two principal components (PC1, PC2). OI = odor intensity, OB = odor briny, OC = odor crustacean, ACI = color intensity, ACU = color uniformity, AEQ = exudate quantity, AET = exudate turbidity, FI = flavor intensity, FB = flavor briny, FC = flavor crustacean, AC = basic taste acid, B = bitter, SW = sweet, SA = salty, U = umami, ATU = aftertaste umami, M = metallic mouth feeling, TH = texture hardness, TCH = chewiness, TCO = cohesiveness, TM = moistness. The colored circles indicate the centroids of the three countries.

The biplot (Figure 2) shows a clear structure in the variable loadings along PC1 and PC2. Texture-related attributes (texture hardness, chewiness, cohesiveness) together with metallic mouth feeling are located in the negative PC1/positive PC2 quadrant, while exudate-related attributes (exudate quantity, exudate turbidity) together with bitter taste and color intensity are positioned in the positive PC1/positive PC2 quadrant. Conversely, odor-related attributes (odor crustacean, odor intensity) together with color uniformity and basic taste acid fall in the positive PC1/negative PC2 quadrant, whereas flavor-related attributes (flavor intensity, flavor briny, flavor crustacean), taste attributes (salty, umami, sweet), aftertaste umami, odor briny and moisture are located in the negative PC1/negative PC2 quadrant.

Examination of the individual scores reveals considerable overlap among the three geographical groups (Italy, Ireland, Norway) along both components, indicating substantial within-group variability relative to the average separation between countries. This is confirmed by comparing the centroids of each group (calculated as the mean of PC1 and PC2 scores for each group’s observations): the Italian centroid is positioned at negative PC1 values (−0.59) and slightly negative PC2 values (−0.27), the Irish centroid lies close to the origin on both axes (PC1 = −0.16; PC2 = 0.06), while the Norwegian centroid shifts towards positive PC1 values (0.75) and slightly positive PC2 values (0.21). This distribution suggests that, on average, Italian samples are more strongly associated with texture-related attributes (hardness, chewiness, cohesiveness) and metallic mouth feeling, whereas Norwegian samples are oriented towards profiles with greater exudate quantity/turbidity, bitterness and color intensity. Irish samples occupy an intermediate position, without a clearly dominant direction along the first two components. Although the score plot suggests a tendency of grouping across geographical origins, the low variance explained by PC1 and PC2 and the substantial overlap among individual observations indicate that intra-group variability far exceeds inter-origin variability, and that centroid differences, while consistent with the environmental and biochemical patterns described below, should be interpreted with caution.

Nutritional analyses revealed several statistically significant differences among mussels from the three geographical origins (Table 3).

Regarding proximate composition, Italian mussels showed higher protein, lipid, carbohydrate, and energy value contents compared with Irish and Norwegian mussels, while exhibiting the lowest moisture levels. Conversely, Irish and Norwegian mussels were characterized by higher moisture contents, whereas Norwegian mussels showed the lowest ash content.

The fatty acid profile also differed among origins. Italian mussels exhibited higher concentrations of several fatty acids, including 15:0, 16:1, 16:1 n-7, 18:1 (including cis and trans isomers), 20:1 (including cis and n-9 isomers), 20:4 n-6 (AA), 20:5 n-3 (EPA), and 22:5 (including n-6 and n-3 isomers). In addition, Italian samples showed higher saturated, monounsaturated, omega-6, and omega-9 fatty acid fractions compared with the other origins.

Norwegian mussels were characterized by higher levels of 18:1t n-7 and 18:4 n-3, while also presenting the lowest concentrations of 16:0 iso and 22:3 n-3. Irish mussels generally showed lower concentrations of several fatty acids, including 12:0, 16:1, 16:1 n-7, 18:1 trans, and 20:5 n-3 (EPA).

No significant differences among origins were observed for several nutritionally relevant fatty acids and lipid fractions, including 18:2 n-6 (LA), 18:3 n-6 (GLA), 18:3 n-3 (ALA), 20:3 n-6 (DGLA), 22:6 n-3 (DHA), polyunsaturated fatty acids (PUFA), and omega-3 fatty acids.

The amino acid profile showed a clear differentiation among the three origins. Italian mussels exhibited higher concentrations of almost all detected amino acids, including aspartic acid, glutamic acid, alanine, arginine, phenylalanine, glycine, isoleucine, histidine, leucine, lysine, proline, serine, tyrosine, threonine, and valine. Total amino acid content was also higher in Italian mussels compared with Irish and Norwegian samples. Hydroxyproline was the only amino acid that did not differ among origins (*p* > 0.05).

Differences were also observed in mineral composition. Irish mussels showed higher iron concentrations compared with the other origins, whereas Italian mussels exhibited higher phosphorus, zinc, and potassium levels. Conversely, no significant differences among origins were detected for copper, selenium, magnesium, and sodium concentrations.

**Figure 3:**
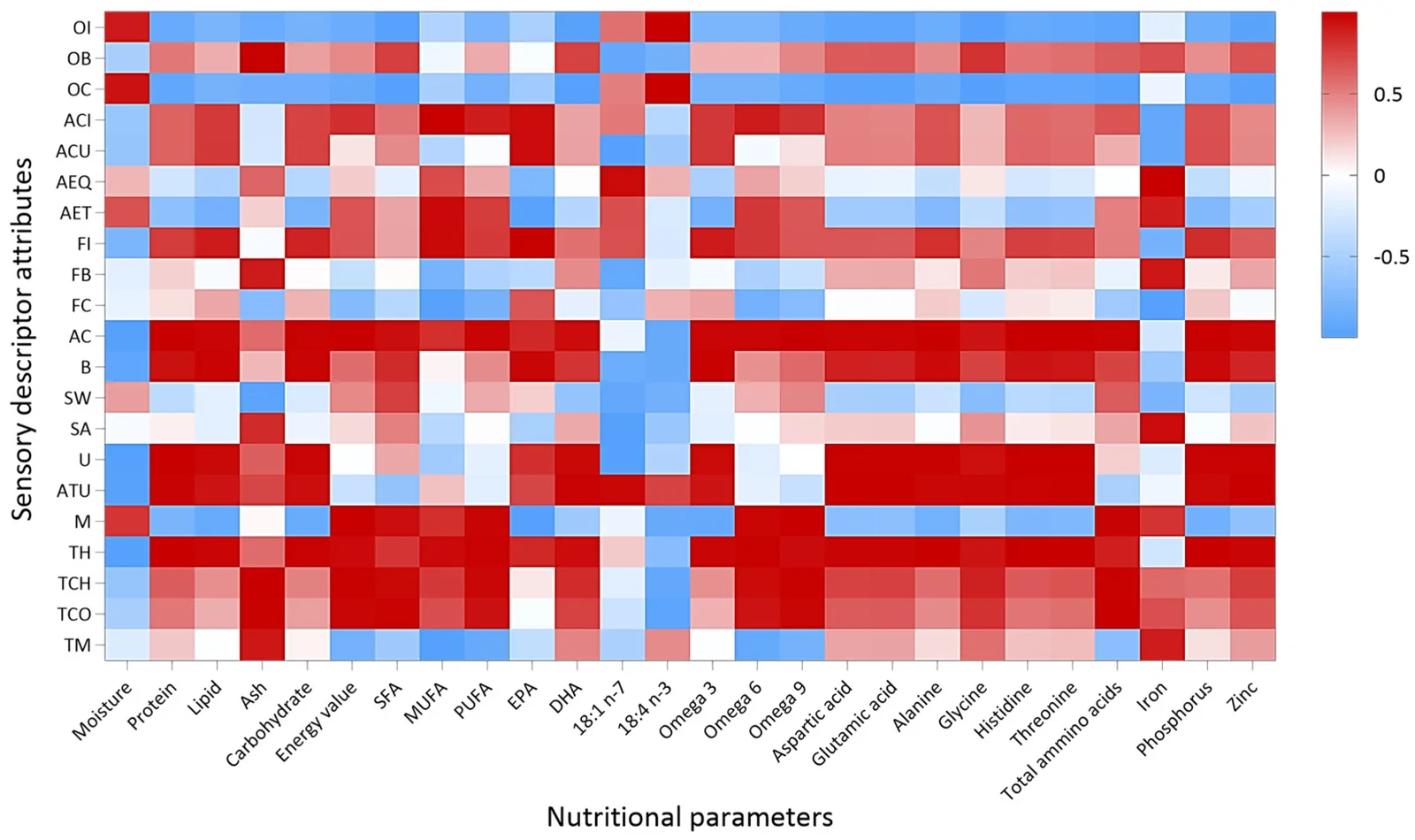
Exploratory correlation heatmap between sensory descriptor attributes and selected nutritional parameters of mussels from Ireland, Norway and Italy. Nutritional variables were selected among those measured based on their relevance to the sensory–nutritional interpretation discussed in the text. Color intensity represents Pearson’s correlation coefficient (r), with red shades indicating positive associations and blue shades indicating negative associations. Correlation coefficients were computed using the three geographical origin means as observations (*n* = 3) and, given the limited number of data points, are intended as descriptive, exploratory indicators of potential macro-trends across origins rather than as statistically validated associations. OI = odor intensity, OB = odor briny, OC = odor crustacean, ACI = color intensity, ACU = color uniformity, AEQ = exudate quantity, AET = exudate turbidity, FI = flavor intensity, FB = flavor briny, FC = flavor crustacean, AC = basic taste acid, B = bitter, SW = sweet, SA = salty, U = umami, ATU = aftertaste umami, M = metallic mouth feeling, TH = texture hardness, TCH = chewiness, TCO = cohesiveness, TM = moistness, SFA = saturated fatty acids, MUFA = monounsaturated fatty acids, PUFA = polyunsaturated fatty acids, EPA = 20:5 n-3 eicosapentaenoic acid, DHA = c22:6 n-3 docosahexaenoic acid.

Exploratory correlation analysis between sensory descriptors and nutritional composition (Figure 3) revealed several notable associations. As these correlations were calculated using only three geographical origin mean values (*n* = 3) as observations, they carry no inferential statistical power and are reported here strictly as descriptive, hypothesis-generating trends rather than statistically validated relationships; the numerical r values are reported only to indicate the direction and approximate magnitude of these exploratory associations. Umami-related descriptors showed positive correlations with amino acid content: umami taste (U) was correlated with glutamic acid (r = 0.993) and aspartic acid (r = 0.994), while aftertaste umami (ATU) showed a positive correlation with glutamic acid content (r = 1.000) and with total omega-3 content (r = 0.921). Bitter taste (B) was also positively correlated with omega-3 fatty acid content (r = 0.992), while salty taste was positively correlated with iron content (r = 0.951). Moreover, briny flavor and briny odor were correlated with iron (r = 0.906 and 0.690 respectively). Texture hardness (TH) showed a negative correlation with moisture content (r = −1.000), consistent with the expected inverse relationship between water content and textural compactness and a positive correlation with protein content (r = 1.000). Exudate quantity showed a light positive correlation with moisture content (r = 0.285). Given the very limited sample size (*n* = 3), these associations should be regarded solely as descriptive patterns, without statistical inference, warranting confirmation in future studies with a larger number of geographical origins and replicated batches.

## 4. Discussion

The present study compared the sensory and nutritional characteristics of mussels (*Mytilus galloprovincialis* and *Mytilus edulis*) sourced from three European farming areas, Italy, Ireland, and Norway, representing different environmental and productive contexts. The aim was to confirm the existence of distinct sensory profiles and nutritional compositions as a function of geographical origin, suggesting a determining role of local environmental conditions in shaping the overall quality of the final product. It should be noted, however, that the Italian samples belong to a different species (*M. galloprovincialis*) than the Irish and Norwegian samples (*M. edulis*). Consequently, part of the differences observed across the three origins may reflect species-specific traits in addition to the environmental factors discussed below.

To interpret these findings coherently, the following discussion adopts a structured causal framework relating to environment, nutritional composition and sensory profile (hereafter, the ENS framework), whereby the environmental conditions of each farming site are first linked to the resulting nutritional composition, which in turn is used to explain the sensory characteristics observed for each origin.

The mussel production cycle varies by country, largely depending on water temperature, nutrient levels, and the specific cultivation method. In Italy and Norway, the cycle lasts roughly 12 months, up to a maximum of 18 months in Norway, whereas in Ireland it typically takes up to 24 months [55,56]. To relate the sensory and nutritional profiles of mussels from the three origins, the environmental conditions of the water in which the animals were farmed were considered over the one-year period preceding harvesting, from July 2024 to July 2025, corresponding to the full farming cycle up to the point of sample collection. It is well known that temperature, salinity, chlorophyll, which indirectly indicates the presence of phytoplankton and therefore food availability for mussels, and current velocity in the water can influence and determine the characteristics of the final commercial product [57].

However, it is important to note that chlorophyll-α, derived from the Copernicus Marine satellite dataset, provides a robust quantitative proxy for phytoplankton biomass and food availability at each site, though it does not resolve the taxonomic composition of the phytoplankton community, and direct water sampling and microalgal characterization were not performed in the present study. Diet composition is known to influence the odor and taste properties of bivalves beyond simple biomass availability, and the three farming areas are known from the independent literature to differ in their typical phytoplankton assemblages: diatom-dominated in the North Adriatic [58], more flagellate/dinoflagellate shifted in Norwegian fjords [59], and periodically enriched in dinoflagellates off Southwest Ireland [60]. Moreover, phytoplankton composition varies considerably across years, seasons, and even short time windows, so site- and time-matched phytoplankton community characterization therefore remains an important direction for future research aiming to clarify the specific dietary drivers of mussel sensory and nutritional quality.

The Northern Adriatic, where the Italian mussels used in this study were farmed, presented the greatest thermal variability (7.98–30.2 °C), which reflects the greater seasonal variability typical of the Mediterranean Sea, slightly higher salinity, and the highest chlorophyll-α peaks (up to 3.37 mg/m^3^), an indicator of greater trophic availability. This is in general accordance with the study of Sani et al. (2026) [61], which documents a strong seasonal cycle of temperature, salinity, and chlorophyll-α in the North Adriatic, with summer temperature peaks of up to 30 °C and high chlorophyll-α values in the northern region influenced by the Po River. The high phytoplankton availability, combined with the moderately high spring/autumn temperatures that accelerate metabolism, stimulates greater synthesis and accumulation of energy reserves in mussels [7,62], thus illustrating how the environment may explain the observed nutritional composition. This is reflected in the data: higher protein content (14.03 g/100 g vs. ~8 g in the other origins), higher lipid content (2.59 g/100 g), higher carbohydrate content (5.8 g/100 g, to be interpreted cautiously as a calculated carbohydrate equivalent), bearing in mind that glycogen represents the primary energy reserve in bivalves [63], lower moisture (74.95%), confirming a more concentrated and dense flesh, as well as higher saturated and monounsaturated fatty acids, higher EPA (0.444 g/100 g), and greater total amino acid content (11.35 g/100 g), particularly glutamic acid, aspartic acid, glycine, and alanine. This proximate and amino acid profile is broadly consistent with other seasonal surveys of *M. galloprovincialis* from Adriatic sites, which similarly report elevated protein, lipid and amino acid contents in warmer, chlorophyll-rich waters [22,64].

Applying the ENS framework, Italian mussels exhibited relatively high sweet taste intensity, without reaching statistical significance among geographical origins. This pattern does not appear to be directly driven by the higher calculated carbohydrate content in Italian samples, since the correlation analysis revealed a negative rather than positive numerical association between sweetness and carbohydrate and also amino acid content. Instead, this dissociation may be explained by the masking effect of Maillard reaction products generated during thermal cooking, which are known to interfere with free sugar- and amino acid-mediated sweet taste perception. Several non-volatile Maillard-derived tastants (e.g., bitter pyridinium and pyranone derivatives formed from reducing sugars and amino acids such as glycine and alanine) can suppress or offset the sweet contribution of the very precursors from which they are formed [65,66].

The high glutamic and aspartic acid content is consistent with the significantly higher umami intensity observed in Italian mussels compared to Norwegian samples, in line with the established role of these amino acids as primary umami-active compounds [65]. This is numerically consistent with the positive r values observed between umami taste and both glutamic acid and aspartic acid content, as well as with the r value close to one between aftertaste umami and glutamic acid, which should be interpreted with caution but are compatible with a particularly persistent umami character in Italian mussels.

Although texture hardness did not differ significantly among origins, the numerically higher hardness recorded in Italian mussels is consistent with their lower moisture and higher protein content. When protein concentration is high and moisture is low, water activity is reduced, which promotes extensive intermolecular protein–protein interactions (like disulfide bonding and hydrogen bonding) rather than protein–water binding. This tight structural network offers greater resistance to physical deformation or biting [67] and textural compactness, with the lack of water acting as a plasticizer that causes the macroscopic structure to become rigid and firmly bound [68].

The higher levels of EPA and monounsaturated fatty acids contribute to more delicate marine aromatic notes, as these lipids serve as precursors of short-chain aldehydes and alcohols characteristic of marine aroma. The flavor and aroma of this food matrix that has been heat-processed, or cooked, might have been affected by the Maillard reaction, which consists of lipid oxidation that degrades unsaturated and saturated fatty acids, generating a wide range of products as volatile and non-volatile compounds, especially aldehydes, alcohols, and ketones, which give characteristic marine aroma [69].

Zinc and phosphorus are known to elicit metallic or astringent notes in other food matrices [70]. However, this was not reflected in the present dataset, where metallic mouthfeel did not differ significantly among origins and, contrary to expectation, showed a negative rather than positive correlation with both minerals. This suggests that mineral-driven metallic perception, if present, may be masked or modulated by other compositional factors not captured in this analysis.

Finally, the significantly higher color intensity recorded in Italian mussels compared to Irish samples is consistent with the greater chlorophyll-α availability documented in the Northern Adriatic. Bivalves accumulate diet-derived carotenoid pigments (e.g., fucoxanthin from diatoms) in their soft tissues, and their pigment content has been shown to closely track ambient phytoplankton density in mussels [71]. The higher trophic availability characterizing the Italian farming site may therefore contribute to the more intense visual coloration observed.

Turning instead to the topic of Irish mussels, the Roaring Water Bay site presented moderate and stable temperatures (9.67–16.7 °C) consistent with North Atlantic water masses, favorable salinity, and adequate trophic availability (chlorophyll-α up to 1.6 mg/m^3^), with considerable current velocity (0.444 m/s). The lower and more stable temperatures limit the accumulation of energy reserves but favor a flesh with high water content and a distinct lipid composition, since stable, low temperatures slow metabolism and reduce the conversion of food into stored glycogen, yielding plump, juicy meat with a higher water content [72]. Moreover, current velocity can directly affect filtration rate, and consequently the food intake and energy budget of bivalves: moderate flow enhances particle delivery to the gills, whereas speeds exceeding the species-specific optimum begin to hydrodynamically inhibit the pumping mechanism itself [73]. At the Irish site, the maximum current velocity recorded was the highest among the three origins and could periodically reduce particle capture efficiency together with an increased energetic cost of counteracting drag. Sustained exposure to strong currents also raises the metabolic cost of byssal attachment and of valve closure to resist dislodgement [74,75]. This suggests a possible trade-off at the Irish site between energy invested in attachment/flow resistance and energy available for reserve accumulation. All these findings may explain the following characteristics observed in Irish mussels: very high moisture (84.17 g/100 g), lower protein and lipid contents, the highest 18:4 n-3 among the three origins (0.0695 g/100 g), a precursor of long-chain omega-3 fatty acids [76] important for cardiovascular health [77], lower total amino acid content with a relatively notable presence of histidine, and exceptionally high iron content (54.5 mg/kg), likely linked to the geochemical characteristics of Atlantic waters and local sedimentology: sediments along the western Irish margin are naturally enriched with iron oxides and terrestrial runoff, and coastal upwelling resuspends bottom sediments, bringing large pulses of dissolved iron into the water column [78,79,80]. The high iron content observed in Irish mussels is correlated numerically with briny flavor and briny odor. However, iron itself is not considered a direct contributor to salty taste perception, but rather to metallic/fishy notes, as discussed further below.

The salty taste could be likely linked to the combined effect of tissue water content (represented by moisture level in mussel tissue) diluting or concentrating dissolved sodium ions, and the integral ionic profile shaped by ambient osmotic conditions at the farming site, consistent with the osmoconforming physiology of bivalves, in which hemolymph and tissue ion concentrations (notably sodium) track the osmotic composition of the surrounding seawater [81,82]. The pronounced salty taste perceived in Irish mussels may also relate to the narrow salinity range recorded at the Irish site, the most stable among the three origins. As osmoconforming bivalves, mussels regulate their internal ionic composition in relation to ambient seawater, and such stable salinity conditions may therefore favor a more consistent tissue sodium profile, enhancing the availability of dissolved ions responsible for salty taste. Moreover, the high moisture level of Irish mussels may have contributed to saltiness by facilitating sodium release and diffusion during mastication, rather than simply diluting ionic concentrations, thereby increasing sodium availability for taste perception. As demonstrated by the studies of Phan et al. (2008) and Kuo and Lee (2014), a higher water content in the matrix increases the release of sodium in the mouth during chewing, especially in the early stages, even before saliva is secreted [83,84].

The moisture, protein and mineral trends are in line with the year-round survey of Irish *M. edulis* by Fernández et al. (2015) [12], which likewise reported high moisture content and marked geographical and seasonal variation in biochemical composition.

Following the structured ENS analysis, the very high moisture content is consistent with the significantly greater exudate release observed in Irish mussels and may contribute to a juicier perceived texture, although the specific sensory moistness attribute did not differ significantly among origins. Flesh with higher free water content releases more liquid upon cooking and results in a more tender and succulent bite [85], as water in mussel soft tissue is distributed across both intracellular and extracellular compartments rather than held within a myofibrillar lattice, closely tied to the animal’s physiological condition [86]. An increase in soft tissue water content is often a compensatory response to the depletion of energy reserves, as glycogen, protein, and lipid stores are metabolized and the resulting loss of dry matter is partly replaced by water uptake, diluting the protein fraction [87,88].

The numerically lower hardness recorded, although not statistically significant, is consistent with a lower protein-to-water ratio: a more hydrated, less protein-dense tissue offers less mechanical resistance to compression and chewing, producing the perceived “watery” mouthfeel [89].

Meanwhile, the lower concentration of carbohydrates and amino acids may theoretically contribute to less pronounced sweet and umami notes [90], consistent with the intermediate umami values observed in Irish mussels (not significantly different from either Italian or Norwegian samples), although sweetness itself did not differ significantly among origins, allowing the saline and marine component to emerge as dominant. In bivalve tissue, sweetness comes not only from carbohydrates but also from glycine and alanine, while umami is mainly attributable to free glutamate and aspartate [91]. When these amino acids are low, both taste dimensions are correspondingly weaker. The presence of 18:4 n-3 (stearidonic acid), a metabolic precursor to EPA and DHA, contributes to the fresh marine character of the aroma through its high lipid oxidation potential, producing volatile compounds typically associated with C6 and C8 aldehydes and alcohols characteristic of fresh, oceanic seafood aroma [92,93,94], consistent with lipid oxidation as the principal source of odorous volatiles across aquatic products overall [95]. Against this reduced sweet/umami background, iron, the only mineral present in considerable amounts in the analyzed tissue, becomes proportionally more relevant to the overall sensory impression and is a key factor in explaining the intensity of marine notes perceived. Iron acts as a dual sensory agent: directly, via its intrinsic metallic taste, mediated partly through trigeminal/chemesthetic pathways [96], and indirectly, as a potent pro-oxidant catalyzing the conversion of lipid hydroperoxides into volatile aldehydes such as hexanal and heptanal during cooking [97,98], jointly producing the characteristic “fishy–metallic” note recognized in seafood.

Examining the mussels sourced from Norgeskjell, Norwegian fjords present the lowest temperatures (3.81–15.8 °C), the lowest salinity (29.5–33.4‰), and intense currents (0.238 m/s), with moderate trophic availability (chlorophyll-α up to 1.69 mg/m^3^). Cold waters and strong currents interact to alter bivalve physiology: low temperatures suppress basal metabolic rates, while hydrodynamic stress increases energetic demand for attachment and filtration [75,99].

The significantly lower ash content (1.97 g/100 g) observed in mussels from this site is likely driven primarily by salinity rather than temperature or hydrodynamics alone. As osmoconformers, mussels regulate cell volume mainly through inorganic ions and free amino acids exchanged with the surrounding medium; under the comparatively low salinity Norwegian conditions, the reduced ionic gradient lowers the inorganic solute pool required for osmotic balance, translating into a lower tissue ash fraction, consistent with experimental evidence in *Perna viridis* [100]. The same osmoregulatory mechanism plausibly explains the comparatively low total amino acid content (6.29 g/100 g) recorded, since free amino acids constitute the principal intracellular osmolyte pool that bivalves adjust in response to ambient salinity: taurine and several non-essential amino acids (aspartate, threonine, serine, glycine, arginine) decline rapidly with decreasing salinity in this species [101].

The highest 18:4 n-3 (stearidonic acid) content among the three origins (0.101 g/100 g) is consistent with cold-water homeoviscous adaptation, the process by which poikilotherms counteract the loss of membrane fluidity due to temperature by increasing the proportion of long-chain PUFA relative to saturated fatty acids in membrane phospholipids [102,103]. This has been directly documented in bivalves, including comparable PUFA enrichment under cold exposure in scallop and *M. edulis* gill membranes [104,105], suggesting the elevated 18:4 n-3 recorded here reflects the same adaptive strategy.

The unusually high 18:1 trans n-7 (trans-vaccenic acid) content may reflect a bacterial origin, as trans-monounsaturated fatty acids are established biomarkers of bacterial activity in marine sediments and bivalve tissues rather than being synthesized de novo [106,107], consistent with environmentally driven bacterial fatty acid variation documented in cold-water *M. edulis* [108].

Among the sensory descriptors, the Norwegian profile centers on bitterness and odor intensity as the two established distinguishing traits. Bitter taste showed a positive numerical correlation with total omega-3 fatty acid content; however, since DHA and EPA concentrations did not differ significantly among origins, this association should be more specifically attributed to stearidonic acid (18:4 n-3), the only omega-3 PUFA significantly enriched in Norwegian mussels. Oxidation of 18:4 n-3 generates a complex mixture of secondary lipid oxidation products (aldehydes, ketones) with documented bitter and off-flavor properties [109]. Bitterness in post-harvest bivalves may also arise from the accumulation of ATP-degradation products such as hypoxanthine, particularly under extended or slower cold-chain transport, as documented in fish and shellfish muscle [110]. However, no quantification of ATP-related catabolites was performed in the present study, and this hypothesis would therefore require biochemical assays to be tested. The same lipid-oxidation-derived volatiles may also contribute to the higher overall odor intensity recorded, since short-chain aldehydes, ketones and alcohols are perceived both retronasally, as taste, and orthonasally, as aroma intensity [95,111]. In addition, dimethyl sulfide (DMS), widely recognized as a principal contributor to the characteristic “smell of the sea,” is produced through microbial breakdown of its precursor dimethylsulfoniopropionate (DMSP), an osmolyte synthesized by marine phytoplankton; DMS/DMSP cycling is particularly intense in cold, nutrient-rich waters such as the Norwegian fjords [112]. The strong currents recorded at this site, by enhancing filtration rate and thus exposure to phytoplankton-derived metabolites [74], may further increase the accumulation of DMS/DMSP-related volatiles and algal secondary metabolites released by grazing-damaged microalgae, plausibly contributing to the higher odor intensity observed and the aromatic complexity of the profile [113]. The greater physical effort demanded by strong currents may also drive a shift toward anaerobic metabolism, leading to the accumulation of succinate and “opine” compounds (octopine, strombine) [114,115]. Succinate is a recognized contributor to umami and broth-like notes in molluscs [116], though given the significantly lower umami recorded in Norwegian compared to Italian mussels, any such contribution appears modest relative to the amino acid-driven mechanism described above.

The numerically softer and more cohesive texture recorded compared to Italian mussels, although not statistically significant, despite a moisture content similar to Irish samples, may be tentatively explained by a different protein structure: low temperatures are associated with fewer stabilizing hydrogen bonds and salt bridges, resulting in a more flexible, less thermally stable structure that yields a more cohesive, but not necessarily harder, flesh [117,118].

The results indicate that environmental conditions at the farming site are consistent with the observed organoleptic differences, outlining regional aromatic signatures that, despite the considerable individual variability within each origin group, align with the environmentally driven mechanisms discussed above [10]. However, because the Italian samples belong to a different species (*M. galloprovincialis*) than the Irish and Norwegian samples (*M. edulis*), part of the differences described above may also reflect species-specific physiological and biochemical traits rather than environmental conditions alone, as mussels of different species reared under common conditions have been reported to differ in biochemical composition [119]. Nevertheless, the comparison between Irish and Norwegian mussels, both belonging to *M. edulis* yet cultivated in contrasting marine environments, provides direct evidence of a true environment-driven effect. Irish mussels, farmed in warmer, more current-swept Atlantic waters with iron-enriched sediments, showed higher moisture, higher iron content, and more pronounced salty/briny notes, whereas Norwegian mussels, farmed in colder, lower-salinity fjord waters, showed the lowest ash and amino acid content and the highest bitterness, linked to their distinctive 18:4 n-3 enrichment. This within-species comparison reinforces the interpretation that farming environment, independent of species identity, is a primary driver of the sensory and nutritional differentiation observed across the origins and strengthens the overall conclusion that geographical/environmental conditions meaningfully shape mussel quality beyond genetic background. Future studies comparing *M. galloprovincialis* and *M. edulis* under shared environmental conditions will further elucidate the relative contribution of genetics versus environment.

It should also be acknowledged that, for each origin, mussels were sampled from a single batch and at a single time point (summer 2025), which allowed a controlled and directly comparable assessment of the three sites under matched harvesting conditions, but does not capture the pronounced seasonal fluctuations known to affect the biochemical composition of mussels [12]. Cross-seasonal sampling at each site, covering at least a full annual cycle, will be necessary in future research to verify the temporal stability of these signatures and to discriminate seasonal from strictly geographical effects.

Factors such as salinity, temperature, hydrodynamic regime, and trophic availability directly influence the biochemical composition of mussel tissues, the concentration of secondary aromatic metabolites, and ultimately the sensory perception of the final product. These findings provide initial evidence supporting the concept of ‘marine terroir’ in bivalve molluscs, building on previous evidence that geographical origin shapes the biochemical and sensory identity of farmed mussels [35,36]. Similarly to what is well established for wine and other agricultural products [34], the perceived quality of mussels emerges from a complex interaction between genetics, environment, and farming management.

To conclude, from a practical standpoint, this evidence carries significant implications, as it allows the initial identification of territorially specific sensory signatures, offering preliminary insight into origin-related sensory tendencies. It should be noted, however, that the substantial intra-group variability and the limited variance explained by the first principal components indicate that this dataset is not directly suitable for developing robust origin traceability classification models. Larger sample sizes and/or targeted discriminant approaches would be needed to formally test and validate origin differentiation in future research.

## Figures and Tables

**Table 1:** Selected descriptors used for the final descriptive profile along with their description.

Attributes	Description
***Odour***	
Odor intensity (OI)	Intensity of odor.
Briny (OB)	Intensity of odor reminiscent of seawater.
Crustacean (OC)	Intensity of characteristic crustacean-like odor.
***Appearance***	
Color intensity (ACI)	Color intensity of mussel flesh, ranging from pale cream/light orange to deep orange, evaluated using Pantone^®^ color chips.
Color uniformity (ACU)	Homogeneity of color distribution within the mussel flesh, without darker areas or discolorations.
Exudate quantity (AEQ)	Quantity of liquid released after cooking the sample.
Exudate turbidity (AET)	Suspended particles in exudate that reduce transparency.
***Flavor***	
Flavor intensity (FI)	Intensity of flavor.
Briny (FB)	Intensity of flavor reminiscent of seawater.
Crustacean (FC)	Intensity of characteristic crustacean-like flavor.
***Basic taste***	
Acid (AC)	Intensity of flavor reminiscent of citric acid.
Bitter (B)	Intensity of flavor reminiscent of caffeine.
Sweet (SW)	Intensity of flavor reminiscent of sugar.
Salty (SA)	Intensity of flavor reminiscent of sodium chloride.
Umami (U)	Intensity of umami flavor reminiscent of glutamic acid.
***Aftertaste***	
Umami (ATU)	Persistence of umami sensation after swallowing.
***Mouthfeel***	
Metallic (M)	Metallic sensation reminiscent of a metal coin or canned products.
***Texture***	
Hardness (TH)	Force required to deform the mussel flesh between the molars.
Chewiness (TCH)	Number of chews required before swallowing the mussel flesh.
Cohesiveness (TCO)	Degree to which the mussel flesh deforms before breaking.
Moistness (TM)	Perception of water released by the mussel flesh during chewing.

**Table 2:** Biometric parameters of mussels used in the test (whole specimen weight, soft tissue weight, length, and condition index; mean ± S.D.) and environmental parameters recorded at the mussel farming sites in Italy, Ireland, and Norway from July 2024 to July 2025. Minimum–maximum values are reported for temperature, salinity, and chlorophyll-a concentration, whereas maximum values are reported for current velocity.

	Italy	Ireland	Norway
**Biometric parameters**			
Whole specimen weight (g)	17.97 ± 1.23	16.05 ± 1.01	16.95 ± 2.94
Soft tissue weight (g)	7.16 ± 0.82	6.32 ± 1.26	6.85 ± 1.38
Length (cm)	6.20 ± 0.43	5.95 ± 0.31	6.39 ± 0.54
Condition index (%)	40.03 ± 3.31	39.30 ± 6.67	40.59 ± 5.84
**Environmental parameters**			
Temperature (°C)	7.98–30.2	9.67–16.7	3.81–15.8
Salinity	32.6–36.5	34.1–35.2	29.5–33.4
Max velocity current (m/s)	0.158	0.444	0.238
Chlorophyll-α (mg/m^3^)	0.235–3.37	0.287–1.6	0.184–1.69

**Table 3:** Proximate composition, fatty acids, amino acid profile and the minerals iron, phosphorus, copper, selenium, zinc, magnesium, potassium, and sodium of the tested mussels from the different countries. Data are mean values ± S.D. *p*-value refers to the significance level obtained from one-way ANOVA testing for differences among the three geographical origins (Italy, Ireland, Norway) for each nutritional parameter. Values in bold and the different superscript letters (a, b, c) within the same row indicate statistically significant differences among origins according to Tukey’s post hoc multiple comparison test (*p* < 0.05). LA = linoleic acid; GLA = gamma-linolenic acid; ALA = alpha-linolenic acid; DGLA = dihomo-gamma-linolenic acid; AA = arachidonic acid; EPA = eicosapentaenoic acid; DHA = docosahexaenoic acid; SFA = saturated fatty acids; MUFA = monounsaturated fatty acids; PUFA = polyunsaturated fatty acids.

Parameters	Italy	Ireland	Norway		
*Proximate composition*	Mean ± S.D.	Mean ± S.D.	Mean ± S.D.	Unit	*p*-value
Moisture	74.955 ± 0.361 ^a^	84.165 ± 0.163 ^b^	84.395 ± 0.078 ^b^	g/100 g	**<0.0001**
Protein	14.035 ± 0.0354 ^b^	8.23 ± 0.382 ^a^	8.01 ± 0.170 ^a^	g/100 g	**0.0003**
Lipid	2.595 ± 0.0778 ^b^	1.52 ± 0.297 ^a^	1.745 ± 0.163 ^a^	g/100 g	**0.0251**
Ash	2.61 ± 0.028 ^b^	2.545 ± 0.163 ^b^	1.97 ± 0.028 ^a^	g/100 g	**0.0124**
Carbohydrate *	5.805 ± 0.445 ^b^	3.54 ± 0.240 ^a^	3.88 ± 0.113 ^a^	g/100 g	**0.009**
Energy value	102.5 ± 0.707 ^b^	61 ± 0.000 ^a^	63 ± 1.414 ^a^	kcal/100 g	**<0.0001**
*Fatty acids*					
10:0	0.001495 ± 0.000	0 ± 0.000	0.00236 ± 0.001	g/100 g	>0.05
12:0	0.00153 ± 0.000 ^b^	0 ± 0.000 ^a^	0.00213 ± 0.000 ^b^	g/100 g	**0.0132**
14:0	0.0645 ± 0.002	0.074 ± 0.014	0.0825 ± 0.006	g/100 g	>0.05
15:0 iso	0 ± 0.00	0.00109 ± 0.001	0 ± 0.000	g/100 g	>0.05
15:0	0.0146 ± 0.001 ^b^	0.00945 ± 0.000 ^a^	0.00875 ± 0.001 ^a^	g/100 g	**0.0205**
16:0 iso	0.003635 ± 0.000 ^b^	0.00335 ± 0.000 ^b^	0 ± 0.000 ^a^	g/100 g	**0.0027**
16:0	0.3925 ± 0.018	0.2625 ± 0.012	0.221 ± 0.021	g/100 g	>0.05
17:0 iso	0.0219 ± 0.000 ^b^	0.01455 ± 0.004 ^ab^	0.0087 ± 0.000 ^a^	g/100 g	**0.0302**
16:1	0.242 ± 0.017 ^b^	0.0575 ± 0.012 ^a^	0.196 ± 0.016 ^b^	g/100 g	**0.0024**
17:0 anteiso	0.00228 ± 0.001	0.002065 ± 0.001	0.002835 ± 0.001	g/100 g	>0.05
17:0	0.0186 ± 0.001 ^b^	0.0103 ± 0.001 ^a^	0.00635 ± 0.001 ^a^	g/100 g	**0.0024**
18:0 iso	0 ± 0.000	0.0044 ± 0.001	0 ± 0.000	g/100 g	>0.05
18:0	0.099 ± 0.003	0.076 ± 0.042	0.04515 ± 0.010	g/100 g	>0.05
18:1	0.1355 ± 0.004 ^b^	0.056 ± 0.016 ^a^	0.083 ± 0.001 ^a^	g/100 g	**0.0073**
18:1 trans	0.0131 ± 0.003 ^b^	0 ± 0.000 ^a^	0.01225 ± 0.000 ^b^	g/100 g	**0.0059**
18:1t n-9	0.0118 ± 0.001 ^c^	0 ± 0.000 ^a^	0.00565 ± 0.000 ^b^	g/100 g	**0.0004**
18:1t n-7	0.00261 ± 0.001 ^a^	0 ± 0.000 ^a^	0.0066 ± 0.000 ^b^	g/100 g	**0.0179**
18:1 cis	0.1222 ± 0.001 ^b^	0.0559 ± 0.015 ^a^	0.0707 ± 0.001 ^a^	g/100 g	**0.0102**
18:1 n-9	0.0332 ± 0.002	0.0319 ± 0.010	0.0332 ± 0.002	g/100 g	>0.05
18:1 n-7	0.089 ± 0.001 ^b^	0.024 ± 0.006 ^a^	0.0375 ± 0.001 ^a^	g/100 g	**0.0006**
18:2 n-6 (LA)	0.04075 ± 0.000	0.03375 ± 0.008	0.0376 ± 0.003	g/100 g	>0.05
20:0	0 ± 0.000	0.00192 ± 0.001	0 ± 0.000	g/100 g	>0.05
18:3	0.0755 ± 0.004	0.05325 ± 0.010	0.0765 ± 0.005	g/100 g	>0.05
18:3 trans	0.01635 ± 0.001 ^c^	0 ± 0.000 ^a^	0.00805 ± 0.000 ^b^	g/100 g	**0.0001**
18:3 cis	0.057 ± 0.003	0.0527 ± 0.010	0.0665 ± 0.005	g/100 g	>0.05
18:3 n-6 (GLA)	0.001835 ± 0.000	0.0011 ± 0.001	0.002095 ± 0.000	g/100 g	>0.05
20:1	0.03655 ± 0.000 ^b^	0.02295 ± 0.002 ^a^	0.019 ± 0.002 ^a^	g/100 g	**0.0019**
18:3 n-3 (ALA)	0.057 ± 0.003	0.0527 ± 0.010	0.0665 ± 0.005	g/100 g	>0.05
18:4 n-3	0.03135 ± 0.002 ^a^	0.0695 ± 0.021 ^ab^	0.101 ± 0.007 ^b^	g/100 g	**0.0264**
20:2 n-6	0.0132 ± 0.004	0.00995 ± 0.005	0.0126 ± 0.001	g/100 g	>0.05
22:0	0.0017 ± 0.002	0.0016 ± 0.001	0 ± 0.000	g/100 g	>0.05
20:3	0.0048 ± 0.001	0.002765 ± 0.000	0.00475 ± 0.000	g/100 g	>0.05
20:3 n-6 (DGLA)	0.00262 ± 0.001	0.00137 ± 0.001	0.00171 ± 0.000	g/100 g	>0.05
22:1	0 ± 0.000	0.00164 ± 0.001	0 ± 0.000	g/100 g	>0.05
20:3 n-3	0.00216 ± 0.000	0.00208 ± 0.001	0.00301 ± 0.000	g/100 g	>0.05
20:4 n-6 (AA)	0.0615 ± 0.001 ^c^	0.02325 ± 0.002 ^b^	0.01475 ± 0.001 ^a^	g/100 g	**0.0002**
24:0	0.00216 ± 0.001	0.00149 ± 0.001	0 ± 0.000	g/100 g	>0.05
20:5 n-3 (EPA)	0.444 ± 0.014 ^b^	0.1745 ± 0.037 ^a^	0.319 ± 0.035 ^b^	g/100 g	**0.0073**
22:3 n-3	0.0052 ± 0.000 ^b^	0.003305 ± 0.001 ^b^	0 ± 0.000 ^a^	g/100 g	**0.013**
22:4 n-6	0.01125 ± 0.001	0.00915 ± 0.003	0.01355 ± 0.001	g/100 g	>0.05
22:5	0.05005 ± 0.002 ^b^	0.02555 ± 0.004 ^a^	0.01605 ± 0.001 ^a^	g/100 g	**0.002**
22:5 n-6	0.0233 ± 0.001 ^b^	0.00615 ± 0.000 ^a^	0.003185 ± 0.000 ^a^	g/100 g	**0.0002**
22:5 n-3	0.02675 ± 0.001 ^b^	0.0194 ± 0.004 ^ab^	0.01285 ± 0.001 ^a^	g/100 g	**0.0206**
22:6 n-3 (DHA)	0.5675 ± 0.013	0.375 ± 0.124	0.2855 ± 0.026	g/100 g	>0.05
Polyunsaturated > C20	0.6345 ± 0.012	0.413 ± 0.130	0.315 ± 0.028	g/100 g	>0.05
Saturated (SFA)	0.622 ± 0.017 ^b^	0.4575 ± 0.064 ^ab^	0.3785 ± 0.040 ^a^	g/100 g	**0.0267**
Monounsaturated (MUFA)	0.4135 ± 0.013 ^c^	0.1375 ± 0.025 ^a^	0.298 ± 0.018 ^b^	g/100 g	**0.0017**
Polyunsaturated (PUFA)	1.3025 ± 0.025	0.78 ± 0.209	0.8815 ± 0.080	g/100 g	>0.05
Omega3	1.149 ± 0.030	0.6965 ± 0.196	0.796 ± 0.075	g/100 g	>0.05
Omega6	0.1545 ± 0.005 ^b^	0.08335 ± 0.012 ^a^	0.0855 ± 0.005 ^a^	g/100 g	**0.005**
Omega9	0.0815 ± 0.004 ^b^	0.05565 ± 0.007 ^a^	0.05785 ±0.004 ^a^	g/100 g	**0.0253**
*Amino acids*					
Aspartic acid	1.31 ± 0.028 ^c^	0.835 ± 0.021 ^b^	0.725 ± 0.021 ^a^	g/100 g	**0.0003**
Glutamic acid	1.545 ± 0.021 ^c^	1.05 ± 0.028 ^b^	0.93 ± 0.014 ^a^	g/100 g	**0.0002**
Alanine	0.618 ± 0.003 ^b^	0.426 ± 0.000 ^a^	0.4365 ± 0.006 ^a^	g/100 g	**<0.0001**
Arginine	0.82 ± 0.028 ^c^	0.515 ± 0.001 ^b^	0.4135 ± 0.009 ^a^	g/100 g	**0.0003**
Phenylalanine	0.5525 ± 0.023 ^b^	0.2865 ± 0.001 ^a^	0.2695 ± 0.015 ^a^	g/100 g	**0.0007**
Glycine	0.88 ± 0.014 ^c^	0.66 ± 0.000 ^b^	0.5135 ± 0.004 ^a^	g/100 g	**<0.0001**
Hydroxyproline	0.0568 ± 0.038	0.0955 ± 0.001	0.0382 ± 0.001	g/100 g	>0.05
Isoleucine	0.483 ± 0.007 ^c^	0.3185 ± 0.005 ^b^	0.2825 ± 0.006 ^a^	g/100 g	**0.0001**
Histidine	0.3045 ± 0.011 ^b^	0.153 ± 0.003 ^a^	0.1435 ± 0.006 ^a^	g/100 g	**0.0003**
Leucine	0.765 ± 0.007 ^c^	0.5165 ± 0.004 ^b^	0.4385 ± 0.013 ^a^	g/100 g	**<0.0001**
Lysine	1.01 ± 0.028 ^b^	0.5165 ± 0.006 ^a^	0.4925 ± 0.019 ^a^	g/100 g	**0.0002**
Proline	0.577 ± 0.035 ^b^	0.3415 ± 0.021 ^a^	0.29 ± 0.030 ^a^	g/100 g	**0.0043**
Serine	0.603 ± 0.024 ^b^	0.415 ± 0.008 ^a^	0.3805 ± 0.013 ^a^	g/100 g	**0.0017**
Tyrosine	0.587 ± 0.031 ^b^	0.322 ± 0.001 ^a^	0.275 ± 0.013 ^a^	g/100 g	**0.001**
Threonine	0.675 ± 0.021 ^b^	0.384 ± 0.000 ^a^	0.356 ± 0.013 ^a^	g/100 g	**0.0003**
Valine	0.5615 ± 0.026 ^b^	0.337 ± 0.003 ^a^	0.3085 ± 0.015 ^a^	g/100 g	**0.0013**
Total	11.3483 ± 0.237 ^c^	7.172 ± 0.071 ^b^	6.2932 ± 0.184 ^a^	g/100 g	**0.0002**
*Minerals*					
Iron	23.55 ± 2.758 ^b^	54.5 ± 2.121 ^c^	13.5 ± 0.000 ^a^	mg/kg	**0.0005**
Phosphorus	1780 ± 169.706 ^b^	1160 ± 56.569 ^a^	1205 ± 106.066 ^a^	mg/kg	**0.0239**
Copper	0.815 ± 0.064	1.2 ± 0.255	0.97 ± 0.000	mg/kg	>0.05
Selenium	0.473 ± 0.052	0.6 ± 0.127	0.3755 ± 0.005	mg/kg	>0.05
Zinc	28.7 ± 1.980 ^b^	13.85 ± 1.344 ^a^	9.8 ± 0.141 ^a^	mg/kg	**0.0017**
Magnesium	955 ± 91.924	920 ± 0.000	710 ± 56.569	mg/kg	>0.05
Potassium	1720 ± 127.279 ^b^	1190 ± 56.569 ^a^	1610 ± 141.421 ^ab^	mg/kg	**0.0374**
Sodium	5810 ± 410.122	6350 ± 353.553	4915 ± 445.477	mg/kg	>0.05

* Carbohydrate content was calculated by difference and should be interpreted as total carbohydrate equivalents (see Materials and Methods for details).

## Data Availability

The original contributions presented in this study are included in the article/Supplementary Materials. Further inquiries can be directed to the corresponding author.

## Supplementary Materials

The following supporting information can be downloaded at: https://www.mdpi.com/article/10.3390/foods15173128/s1. Table S1: Complete set of mean values, standard deviations and post-hoc *p*-values for all 21 sensory descriptors.

## Abbreviations

The following abbreviations are used in this manuscript:

QDA^®^	Quantitative Descriptive Analysis	U	Basic taste umami
O	Odor	ATU	Aftertaste umami
F	Flavor	M	Mouthfeel metallic
T	Texture	TH	Texture hardness
A	Appearance	TCH	Texture chewiness
Bt	Basic taste	TCO	Texture cohesiveness
At	Aftertaste	TM	Texture moistness
Mf	Mouthfeel	PCA	Principal component analysis
OI	Odor intensity	LA	Linoleic acid
OB	Odor briny	GLA	Gamma-linolenic acid
OC	Odor crustacean	ALA	Alpha-linoleic acid
ACI	Color intensity	DGLA	Dihomo-gamma-linolenic acid
ACU	Color uniformity	AA	Arachidonic acid
AEQ	Exudate quantity	EPA	Eicosapentaenoic acid
AET	Exudate turbidity	DHA	Docosahexaenoic acid
FI	Flavor intensity	SFA	Saturated fatty acid
FB	Flavor briny	MUFA	Monounsaturated fatty acid
FC	Flavor crustacean	PUFA	Polyunsaturated fatty acid
AC	Basic taste acid	ENS	Environment–Nutritional composition–Sensory profile framework
B	Basic taste bitter	DMS	Dimethyl sulfide
SW	Basic taste sweet	DMSP	Dimethyl sulfoniopropionate
SA	Basic taste salty		
