# Supplementary material for: Sensory and Nutritional Profiling of Commercial Mussels (Mytilus galloprovincialis and Mytilus edulis) Across Different European Marine Origins
Source: Foods. 2026 Sep 3;15(17):3128. doi: 10.3390/foods15173128 (PMC13564763; doi:10.3390/foods15173128)
Supplement: Supplementary file 1 [file foods-15-03128-s001.zip › foods-4504772-supplementary.pdf]

Supplementary Materials

Table S1. Complete set of mean values, standard deviations and post-hoc p-values for all 21 sensory descriptors.

Data are mean values ± S.D. *P-value* refers to the significance level obtained from one-way ANOVA testing for differences among the three geographical origins (Italy, Ireland, Norway) for each sensory descriptor. Values in bold and the different superscript letters (a,b) within the same row indicate statistically significant differences among origins according to Tukey's post-hoc multiple comparison test ($p < 0.05$).

Sensory descriptor attributes	*Italy*	*Ireland*	*Norway*	*P-value*
**ODOUR**				
Odour intensity	49.56±3.95	56.00±6.75	60.67±2.68	>0.05
Briny	33.89±7.44	33.83±7.64	25.78±0.96	>0.05
Crustacean	42.11±5.85	51.17±8.67	56.72±9.32	>0.05
**APPEARANCE**				
Colour intensity	70.61±6.00^{a}	48.28±20.07^{b}	67.06±23.94^{ab}	**0.042**
Colour uniformity	74.06±12.52	62.78±11.98	71.50±5.73	>0.05
Exudate quantity	63.89±17.24^{ab}	84.89±8.47^{a}	58.28±26.51^{b}	**0.016**
Exudate turbidity	78.22±9.64	82.17±10.52	79.11±22.71	>0.05
**FLAVOUR**				
Flavour intensity	56.67±10.37	49.06±7.00	53.67±12.22	>0.05
Briny	50.94±7.04^{ab}	58.06±11.57^{a}	39.11±8.35^{b}	**0.037**
Crustacean	47.28±6.40	39.50±7.11	50.94±6.28	>0.05
**BASIC TASTE**				
Salty	38.56±13.10^{a}	48.56±5.35^{a}	27.72±8.64^{b}	**<0.001**
Sweet	19.17±16.62	18.22±2.59	24.33±7.49	>0.05
Acid	1.94±3.37	0.00±0.00	0.17±0.29	>0.05
Bitter	18.06±27.96	2.72±2.02	7.78±7.56	>0.05
Umami	59.28±7.75	46.11±8.03	45.39±7.11	>0.05
**AFTERTASTE**				
Umami	62.56±5.11^{a}	46.83±12.26^{ab}	42.94±12.01^{b}	**0.030**
**MOUTHFEEL**				
Metallic	16.61±7.19	21.94±4.32	19.06±4.70	>0.05
**TEXTURE**				
Hardness	26.39±5.39	23.28±5.48	22.89±8.54	>0.05
Chewiness	28.83±3.00	27.56±8.33	22.33±10.11	>0.05
Cohesiveness	25.33±2.42	24.94±9.73	19.56±8.19	>0.05
Moistness	36.89±5.26	38.94±10.25	32.56±11.07	>0.05
